# Management of people with acute low-back pain: a survey of Australian chiropractors

**DOI:** 10.1186/2045-709X-19-29

**Published:** 2011-12-15

**Authors:** Bruce F Walker, Simon D French, Matthew J Page, Denise A O'Connor, Joanne E McKenzie, Katherine Beringer, Kerry Murphy, Jenny L Keating, Susan Michie, Jill J Francis, Sally E Green

**Affiliations:** 1 School of Chiropractic and Sports Science, Murdoch University, Murdoch, 6150, Western Australia; 2 School of Public Health and Preventive Medicine, Monash University, Level 6, The Alfred Centre, 99 Commercial Road, Melbourne, Victoria 3004, Australia; 3 Primary Care Research Unit, University of Melbourne, 200 Berkeley St, Carlton, Victoria, 3010, Australia; 4 Department of Physiotherapy, School of Primary Health Care, Faculty of Medicine Nursing and Health Science, Monash University, PO Box 527 Frankston, Victoria, 3199 Australia; 5 Centre for Outcomes Research and Effectiveness, Department of Clinical, Educational and Health Psychology, University College London, WC1E 7HB, London, UK; 6 Health Services Research Unit, Health Sciences Building, University of Aberdeen AB25 2ZD, UK

## Abstract

**Introduction:**

Chiropractors commonly provide care to people with acute low-back pain (LBP). The aim of this survey was to determine how chiropractors intend to support and manage people with acute LBP and if this management is in accordance with two recommendations from an Australian evidence-based acute LBP guideline. The two recommendations were directed at minimising the use of plain x-ray and encouraging the patient to stay active.

**Methods:**

This is a cross sectional survey of chiropractors in Australia. This paper is part of the ALIGN study in which a targeted implementation strategy was developed to improve the management of acute LBP in a chiropractic setting. This implementation strategy was subsequently tested in a cluster randomised controlled trial. In this survey phase of the ALIGN study we approached a random sample of 880 chiropractors in three States of Australia. The mailed questionnaire consisted of five patient vignettes designed to represent people who would typically present to chiropractors with acute LBP. Four vignettes represented people who, according to the guideline, would not require a plain lumbar x-ray, and one vignette represented a person with a suspected vertebral fracture. Respondents were asked, for each vignette, to indicate which investigation(s) they would order, and which intervention(s) they would recommend or undertake.

**Results:**

Of the 880 chiropractors approached, 137 were deemed ineligible to participate, mostly because they were not currently practising, or mail was returned to sender. We received completed questionnaires from 274 chiropractors (response rate of 37%). Male chiropractors made up 66% of respondents, 75% practised in an urban location and their mean number of years in practice was 15. Across the four vignettes where an x-ray was not indicated 68% (95% Confidence Intervals (CI): 64%, 71%) of chiropractors responded that they would order or take an x-ray. In addition 51% (95%CI: 47%, 56%) indicated they would give advice to stay active when it was indicated. For the vignette where a fracture was suspected, 95% (95% CI: 91%, 97%) of chiropractors would order an x-ray.

**Conclusion:**

The intention of chiropractors surveyed in this study shows low adherence to two recommendations from an evidence-based guideline for acute LBP. Quality of care for these patients could be improved through effective implementation of evidence-based guidelines. Further research to find cost-effective methods to increase implementation is warranted.

## Introduction

Low back pain (LBP) is a common and costly problem in high income countries like Australia. At any one time, approximately one in five Australians has LBP, and four out of five Australians will experience it at some time in their lives [1]. The direct and indirect cost of LBP in 2001 was estimated to total AUD$9,175 million [2]. Chiropractors provide a significant proportion of the care for people with LBP in Australia [3].

In 2004, an evidence-based clinical practice guideline for acute LBP [4] was sent to all primary healthcare providers in Australia, including chiropractors. The guideline provided evidence-based recommendations for the diagnosis, prognosis and treatment of acute non-specific LBP in primary care settings. Two relevant key messages were (i) that plain x-rays of the lumbar spine are not routinely recommended for people with acute non-specific LBP as they are of limited diagnostic value and provide no benefits in pain, function or quality of life, and (ii) advising these patients to stay active produces a beneficial effect on pain, rate of recovery and function [4]. Although the guideline was released in 2004, more recent systematic reviews of randomised trials still support these two recommendations [5,6], and a review of LBP guidelines showed that more recent guidelines made similar recommendations [7].

This paper reports on one phase of the ALIGN study. The ALIGN study explored barriers and enablers to the uptake of the Australian acute LBP guideline in order to develop a targeted implementation strategy for use in a chiropractic setting which was subsequently tested in a cluster randomised controlled trial [8]. Other aspects of the ALIGN study will be reported elsewhere. The aim of this part of the study was to determine the intended practices of chiropractors in the management of acute LBP and whether they are in accordance with two recommendations in the Australian evidence-based acute LBP guideline [4] and more generally how chiropractors intend to support or manage people with acute LBP.

## Methods

### Design

This study is a cross sectional survey of chiropractors in Australia.

### Sample size and participants

The sample size was calculated to address the primary aim of the survey which was to identify factors (e.g. knowledge) that were predictive of intention to perform a particular behaviour, and then to use this information to develop the ALIGN implementation strategy. The sample size was calculated to detect a 0.5 difference in intention to perform a particular behaviour, measured on a 7-point Likert scale, between dichotomised factors (assuming an equal distribution of participants in each dichotomy), with 90% power. To detect this difference, a sample of 440 chiropractors was required, assuming a 5% significance level and standard deviation of 1.6 (estimated from a similar survey our study group had undertaken with general medical practitioners [9]). We allowed for a 50% response rate, and therefore approached 880 chiropractors to participate.

Chiropractors were randomly sampled from three strata, defined by States in Australia, with the same proportion of chiropractors approached in each state. The entire sample frame of 1760 was obtained from the Chiropractic Registration Boards. The numbers approached in Victoria, South Australia, and Western Australia were, respectively, 553, 165, and 162. These States were chosen as other Australian States' Registration Boards would not release contact details of their registrants for research purposes.

### Survey instrument

Five patient vignettes (Additional File 1) were adapted from another study in a general medical practice setting [10]. The vignettes were designed to reflect people with acute LBP who would typically present to chiropractors. Elements of each vignette were drawn from the acute LBP guideline, from another study in Victoria that evaluated a media campaign for LBP [11] and from the North-East X-ray Utilisation (NEXUS) study, which evaluated the effectiveness of audit and feedback and educational reminders on United Kingdom general practitioners' ordering of lumbar spine and knee x-rays [12]. The chiropractor's role and patient elements that were included in each vignette were designed to contextualise the vignettes. These elements were chosen because they have been recognised in previous research as potential barriers to practitioners making decisions consistent with guideline recommendations [12].

Four of the five vignettes represented people who would not require a plain lumbar x-ray and would benefit from being advised to stay active as per the recommendations from the Australian guideline [4], and one vignette represented someone with a suspected vertebral fracture who would require a plain x-ray. From a selection of response options respondents were asked to indicate which investigation(s) they would order for the patient described in each vignette, and which intervention(s) they would recommend or undertake (see Additional File 2 for options given). The investigations or treatments offered as options in the responses were developed by two experienced chiropractors who were investigators on the project team. They were chosen by the chiropractic investigators (BW and SF) as those investigations or treatments most likely to be used or recommended by chiropractors. Chiropractor respondents also had three "other" response options and could write free text to describe their intention. The five patient vignettes were pre-tested in a pilot study using six (of eight randomly sampled) Victorian chiropractors. These chiropractors had previously been interviewed about LBP management in a separate phase of the ALIGN study [8]. The pilot results led to minor changes.

### Survey administration

The Modified Total Design Method [13] was used for chiropractor recruitment. The sample initially received an invitation letter to participate in the study. This letter was accompanied by a plain language statement, consent form, and reply-paid envelope (for returning an individual's decision to opt-in [or opt-out] by mail). A reminder letter was sent to non-respondents every two weeks for up to a total of eight weeks after the initial letter was sent. Both the fourth and eighth week reminder letters were accompanied by a plain language statement, consent form, survey document and reply-paid envelope.

The Total Design Method usually includes the use of a return mail card to signify that the respondent has opted-in to the study. The return mail card can also be used by individuals to indicate that they do not wish to participate in the study. In this study we replaced the return mail card with an option on the consent form for individuals to indicate they did not wish to participate (i.e. opt-out) in the study (and not be contacted further about this study). It was therefore considered that a return mail card, as per the Total Design Method, was not necessary in this study.

### Analysis

Double data entry was performed independently by two researchers (MP and KB) and discrepancies were resolved via discussion with a third researcher.

Descriptive statistics were used to describe demographic details of the chiropractic respondents. The percentage of chiropractic respondents indicating which investigation(s) and which intervention(s) they would undertake for each vignette was calculated. Confidence intervals were calculated adjusting for the stratification variable. Exact binomial confidence intervals were calculated when the normal approximation was not valid [14]. Confidence intervals calculated for the percentage of respondents indicating which investigation(s) and which intervention(s) they would undertake across multiple vignettes (e.g. recommending advice to stay active across vignettes 1 to 4) were adjusted to allow for the correlation of responses within chiropractor. Analyses were undertaken using the svy commands in the statistical package Stata (StataCorp. 2007. Stata Statistical Software: Release 10.1. College Station, TX: StataCorp LP) with Taylor series approximations for the estimation of standard errors.

Human research ethics approval for this study was granted by Monash University (CF07/1060 - 2007000274).

## Results

The survey was administered in April 2009. One-hundred and thirty seven (16%) chiropractors approached were deemed ineligible to participate, mostly because they were not currently practising, or mail was returned to sender. We received completed questionnaires from 274 of 743 eligible chiropractors, resulting in a response rate of 37%. Male chiropractors made up 66% of respondents, 75% practised in an urban location and their mean number of years in practice was 15 (Table 1).

**Table 1:** Demographic details of chiropractor survey respondents

Variable	Category	Chiropractors (N = 274)
Sex (n, %)	Male	181 (66)
Age (Mean, SD) (Median, IQR)		41 (12) 40 (32, 49)
Practice location (n, %)	Urban	206 (75)
	Rural	64 (23)
	Remote	4 (1)
Practice type (n, %)	Group	173 (63)
	Solo	101 (37)
Years in clinical practice (Mean, SD) (Median, IQR)		15 (11)**^†^** 13 (7, 21)
Hours practicing/week (Mean, SD) (Median, IQR)		30 (12) 30 (25, 35)
Total patients/week (Mean, SD) (Median, IQR)		104 (69)**^‡^** 90 (60, 140)
Acute LBP patients/week (Mean, SD) (Median, IQR)		23 (24)* 15 (5, 30)
Involvement in teaching (n, %)	Yes	33 (12.1)
Formal postgraduate training relevant to LBP (n, %)	Yes	96 (35)**^†^**
Access to bulk-billing radiology service (n, %)	Yes	245 (89)
Take own x-rays (n, %)	Yes	52 (19)
Gonstead practitioner (n, %)	Yes	31 (11)

SD = standard deviation; IQR = interquartile range; LBP = low back pain; Gonstead practitioner: A type of chiropractor known to use plain x-ray more frequently.

^† ^N = 273; ^‡ ^N = 269; * N = 264

Across the four vignettes where an x-ray was not indicated according to the guideline, 68% (95% CI: 64%, 71%) of chiropractors responded that they would order or take an x-ray, and 51% (95% CI: 47%, 56%) indicated they would give advice to stay active. For the vignette where a fracture was suspected, 95% (95% CI: 91%, 97%) of chiropractors would order an x-ray (Table 2). In addition, across all five vignettes, 25% (95% CI: 20%, 29%) of chiropractors would take, or order, a full spine plain x-ray.

**Table 2:** Percentage (95% confidence interval) of chiropractic respondents ordering, undertaking, or recommending investigations

Investigation	Vignette 1		Vignette 2		Vignette 3		Vignette 4		Vignette 5*	
N = 274										
Lumbosacral plain x-ray	40	(34, 46)	52	(46, 58)	47	(41, 53)	40	(34, 46)	67	(62, 73)
Lumbosacral CT scan	0	(0, 2)**^†^**	2	(1, 4) **^†^**	18	(13, 22)	10	(7, 14)	4	(2, 7) **^†^**
Lumbosacral MRI	1	(0, 3) **^†^**	1	(0, 4) **^†^**	9	(5, 12)	4	(2, 7) **^†^**	2	(1, 5) **^†^**
Full spine plain x-ray	24	(19, 29)	32	(27, 38)	14	(10, 18)	25	(20, 30)	28	(23, 33)
None	34	(28, 39)	14	(9, 18)	20	(15, 25)	28	(23, 34)	3	(1, 6) **^†^**
Overall x-ray guideline adherence	37	(31, 43)	16	(12, 20)	39	(33, 45)	37	(31, 43)	95	(91, 97) **^†‡^**

* The only vignette where guideline recommends plain x-ray and not advice to stay active

Note: For Vignettes 1-4, x-ray guideline adherence is defined as the number of participants not ordering any type of x-ray (e.g., lumbosacral, full spine, AP lateral, pelvic)

Note: For Vignette 5, x-ray guideline adherence is defined as the number of participants ordering some type of x-ray (e.g., lumbosacral, full spine, AP lateral, pelvic)

† Exact binomial confidence calculated [14]

‡ Based on 273 participants.

Regarding treatment and management options that the chiropractors would employ 76% (95% CI: 72%, 79%) of chiropractors indicated that they would perform spinal manipulation/adjustment for vignettes 1 to 4 (range 63% to 92%), and 51% (95%CI: 47%, 56%) of chiropractors indicated they would give advice to stay active when it was indicated. Other treatments and care that chiropractors indicated they would provide for each vignette are detailed in Table 3. Around one third of chiropractors surveyed (35%; 95% CI: 29%, 40%) indicated that they would perform spinal manipulation of the spine in a patient with a likely fracture for vignette 5.

**Table 3:** Interventions undertaken or recommended by chiropractic respondents - n (%)

Response options	Vignette 1	Vignette 2	Vignette 3	Vignette 4	Vignette 5
Bed rest*	1 (0)	1 (0)	3 (1)	47 (17)	36 (13)
Bed rest outside guideline recommendations^†^	0 (0)	0 (0)	2 (1)	12 (4)**^‡^**	NA
Paracetamol	18 (7)	21 (8)	18 (7)	53 (19)	99 (36)
NSAIDs	38 (14)	21 (8)	65 (24)	125 (46)	48 (18)
Back exercises	79 (29)	97 (35)	133 (49)	41 (15)	14 (5)
General exercise	135 (49)	213 (78)	130 (47)	94 (34)	62 (23)
Advice to stay active	143 (52)	183 (67)	134 (49)	101 (37)	68 (25)
Advice regarding alternate ways of moving	184 (67)	168 (61)	141 (51)	169 (62)	131 (48)
Advice to avoid pain provoking movements	196 (72)	181 (66)	134 (49)	191 (70)	155 (57)
Work modification	105 (38)	113 (41)	119 (43)	132 (48)	28 (10)
Spinal manipulation/adjustment	251 (92)	219 (80)	189 (69)	173 (63)	95 (35)
Mobilisation	84 (31)	96 (35)	66 (24)	93 (34)	55 (20)
Massage	155 (57)	162 (59)	120 (44)	131 (48)	106 (39)
Lumbar supports	10 (4)	10 (4)	29 (11)	42 (15)	23 (8)
Spinal traction	6 (2)	5 (2)	21 (8)	23 (8)	4 (1)
Acupuncture	15 (5)	13 (5)	29 (11)	22 (8)	11 (4)
Electrotherapy	22 (8)	20 (7)	24 (9)	31 (11)	25 (9)
Thermal modalities	135 (49)	105 (38)	74 (27)	151 (55)	120 (44)
Printed information	29 (11)	29 (11)	15 (5)	19 (7)	4 (1)
Referral to another health care provider	6 (2)	25 (9)	84 (31)	28 (10)	35 (13)

N = 274 chiropractors unless otherwise specified.

* Advising bed rest (irrespective of the number of days).

^† ^Advising bed rest for more than 2 days.

**^‡ ^**N = 268

## Discussion

The aim of this survey was to determine if management intended by chiropractors is in accordance with two key recommendations in the Australian evidence-based acute LBP guideline [4] and more generally how chiropractors intend to support or manage people with acute LBP. Ours is the first study undertaken in Australia, that we are aware of, that has investigated these two key recommendations for people with acute LBP in chiropractic practice.

### X-ray usage

Where an x-ray was not recommended according to the guideline 68% of chiropractors responded that they would order or take an x-ray, indicating an overall x-ray guideline adherence of 32%. It has been reported previously that chiropractors use plain x-rays at a high frequency for people with acute non-specific LBP, with rates varying from 12% to 63% [15-22]. Our findings are consistent with these studies and suggest that a high proportion of chiropractors who responded to this survey are not complying with the guideline on the use of plain x-ray for acute LBP. Some chiropractors order or take plain x-rays to rule out pathology, screen for contraindications to manipulation, assist in the selection and delivery of treatment, and monitor patient progress [23]. A post-hoc review of the literature found no substantive evidence of harm from chiropractic manipulation because plain x-ray had not been used nor enhanced efficacy.

Across all five vignettes approximately 25% (95% CI: 20%, 29%) of chiropractors would take, or order, a full spine plain x-ray. This was despite the lack of information in the vignettes about any symptoms or examination findings at the other regions of the spine. There is no evidence that full spine x-rays are warranted in these vignettes and this finding is troubling considering the radiation dose that the patient would receive [24,25].

Around one third of chiropractors surveyed (35%; 95% CI: 29%, 40%) would perform spinal manipulation of the spine in a patient with a likely fracture. We did not investigate the reasons for this, however it may be that a) the potential for fracture diagnosis in the vignette was not considered, or b) the chiropractor intended to apply manipulation to regions above or below the fracture site.

The findings above have implications for patients' safety and health resource use. First, a full spine x-ray will expose the patient to needless ionising radiation, and manipulation of a spinal fracture may worsen the fracture with potentially serious consequences. Second, the findings have implications for third party payments for these extensive and expensive diagnostic imaging.

### Giving advice to stay active

Over half the chiropractors (51%; 95% CI: 47%, 56%) indicated that in the first four vignettes they would advise the patient to stay active, while 60% (95% CI: 57%, 64%) responded they would give exercises (back specific or general) to people seen who were similar to the first four vignettes. In this sample of chiropractors, there appears to be a need to encourage the giving of advice to patients with acute LBP to stay active, regardless of any exercises they would recommend to the patient in order to bring chiropractic management in line with evidence-based practice.

### Study limitations

The response rate was low (37%) and therefore the results have an increased potential for selection bias where those who choose to participate differ in important characteristics with those who do not choose to participate [26]. It may be speculated that those who responded were more likely to comply with the guideline, in which case we have calculated underestimates of guideline compliance. If this is the case the problem may be greater than our results demonstrate. It also may be speculated that those who responded were less likely to comply with the guideline, in which case we have calculated overestimates of guideline compliance. Regardless, we can only conclude for the respondents to this survey and are not able to generalise to the broader population of chiropractors in these three States of Australia. No data were available for the broader chiropractic population at the time we undertook this study, so we were unable to compare the characteristics of the respondents to the characteristics of the broader Australian chiropractic population to investigate non-response bias. The response rate is lower than those seen in other surveys of health professionals where a mean of 58% has been reported, however it has also been demonstrated that response rates of health professionals are decreasing over time [26]. Recently published surveys of chiropractors have reported response rates ranging from 38% to 88% [27-31].

Patient vignettes are limited by a lack of detail that may be gleaned during a patient consultation, including visual cues relating to pain during an examination. Further, patient vignettes may not be sensitive enough to pick up the fine nuances of practice that would better guide the practitioner [32]. Therefore, our conclusions are based on a measure of proxy behaviour rather than real behaviour. More research is needed to establish the extent to which proxy measures of behaviour can predict actual behaviour.

## Conclusion

The current intention of a significant number of Australian chiropractors who responded to this survey and who manage people with acute LBP is not in accordance with evidence-based recommendations. Quality of care for these patients could potentially be improved through effective implementation of evidence-based guidelines. Further research to find cost-effective methods to increase implementation is warranted.

## Competing interests

Bruce Walker is Editor in Chief and Simon French is an Associate Editor of Chiropractic & Manual Therapies. All decisions on this manuscript were made by another editor. All other authors declare that they have no competing interests.

## Authors' contributions

All authors contributed to the design and conduct of the study. SF wrote the first draft of this manuscript with comments from BW. JM undertook the statistical analysis. BW undertook further revisions of the manuscript with feedback from all authors. All authors agreed to the final manuscript.

## Supplementary Material

Additional file 1 **Patient vignettes**. This file contains the five patient vignettes that were used in the survey. Click here for file

Additional file 2 **Investigations and intervention options for the five patient vignettes**. This file shows the options available to the survey respondents for investigations and interventions for each of the patient vignettes. Click here for file
