# Supplementary material for: Management of people with acute low-back pain: a survey of Australian chiropractors
Source: Chiropr Man Therap. 2011 Dec 15;19:29. doi: 10.1186/2045-709X-19-29 (PMC3265419; doi:10.1186/2045-709X-19-29)
Supplement: Additional file 1 — Patient vignettes. This file contains the five patient vignettes that were used in the survey. [file 2045-709X-19-29-S1.PDF]

Additional File 1. Patient vignettes

Survey introduction for chiropractors and the five vignettes

This section contains five hypothetical vignettes about patients who present to you with acute low-back pain. In the vignettes we have varied a range of features that might influence your management decisions (in regards to investigations you might order and interventions you might recommend or undertake). At the end of each vignette we ask you to indicate what investigation(s) you would order for the patient described in the vignette, and what intervention(s) you would recommend or undertake for this patient. We are aware that the vignette format means that skills you may normally draw on, such as evaluating non-verbal cues from the patient and performing a physical examination, cannot be a factor in your assessment. Nevertheless, given this understanding, we hope that you address each vignette and answer the questions as best as you can with the information provided. We have left space for you to comment on your decisions, if you wish.

Vignette 1
A 48 year old office worker attends your clinic. He is usually very active, playing lots of sport and doing regular exercise (e.g. jogging, gym). He has low-back pain, rated 5 out of 10. The pain started two weeks ago and is located in the low-back region, right sided, no radiation. The pain is relieved by stretching his low-back and using a heated wheat bag. The pain is worse after playing sport, to the point where in the last week he had to stop mid-game during basketball. He has no previous history of low-back pain. The patient thinks that an x-ray is required to "find out what is wrong", and he is fearful that movement and activity might make the pain worse.

Vignette 2
A 57 year old office worker sees you for low-back pain. She says her pain began 8 weeks ago. There was no specific incident that caused the pain. The pain is located in the lower back region, with no radiation. The pain is a dull ache (3 out of 10), with occasional sharp "twinges" with certain movements. The pain is relieved by heat and a massage from her spouse. She has no history of low-back pain. The patient is overweight (BMI 30), has mild hypertension, and a family history of type 2 diabetes. The patient rarely does any exercise. During the consultation she indicates to you that she is anxious that she may have a serious disease. The patient says "a friend had low-back pain like this and they had an x-ray and it showed that they really had something major wrong with them". She repeatedly requests an x-ray during the consultation.

Vignette 3
A 36 year old real estate agent consults you for his low-back pain. He comes in on a very busy day at the practice and there are many patients already in the waiting room wanting to see you. The pain has been present for six weeks, starting two days after moving heavy furniture at home. The pain is described as an ache (4 to 5 out of 10). There is no radiation. He has had previous, similar episodes of low-back pain that have lasted one to two weeks. The patient has no other health concerns. The patient has seen you weekly over the last four weeks for his low-back pain and complains that it is not improving. He is frustrated with his lack of improvement and thinks something different needs to be done. He is dissatisfied

that he has not already been referred for further investigation, and insists that you refer him now.

Vignette 4
A 28 year old woman has suffered from low-back pain for a week. She has been unable to do her job managing a hospital cafeteria for this time. She walks slowly into your consultation room, holding her back and grimacing. You notice her abnormal posture. She sits with a loud groan. She says she has severe low-back pain, describing it as 9 out of 10. While anxious to return to work, she feels immobilised by the pain. There is no history of trauma. The pain is in the low-back area, without radiation. On physical examination there is marked limitation of anterior flexion and tenderness in the left paraspinal region. The neurological examination is normal with straight leg raising to 90 degrees. She has had numerous episodes of back pain in the past but thinks this is the worst episode she has ever had and is very worried that whatever is causing her problem is getting worse.

Vignette 5
A 67-year-old woman attends your clinic. She has low-back pain that started immediately after she fell off a chair at home 4 days ago while reaching for her glasses. The pain has been constant since then, although she gets some slight relief from paracetamol.
