# Supplementary material for: Management of people with acute low-back pain: a survey of Australian chiropractors
Source: Chiropr Man Therap. 2011 Dec 15;19:29. doi: 10.1186/2045-709X-19-29 (PMC3265419; doi:10.1186/2045-709X-19-29)
Supplement: Additional file 2 — Investigations and intervention options for the five patient vignettes. This file shows the options available to the survey respondents for investigations and interventions for each of the patient vignettes. [file 2045-709X-19-29-S2.PDF]

Additional File 2. Investigations and intervention options for the five patient vignettes

1. Which investigations would you order, undertake or recommend for this patient at this visit? *Please tick all that apply:*

- ☐ Lumbosacral plain x-ray
- ☐ Lumbar CT scan
- ☐ Lumbar MRI
- ☐ Full spine plain x-ray
- ☐ Other 1 (*please specify*) ___________________________
- ☐ Other 2 (*please specify*) ___________________________
- ☐ Other 3 (*please specify*) ___________________________
- ☐ None

2. Which interventions would you recommend or undertake for this patient at this visit? *Please tick all that apply:*

- ☐ Bed rest for ____ days (*please specify number of days*)
- ☐ Paracetamol
- ☐ Non-steroidal anti-inflammatory drugs (NSAIDs)
- ☐ Back exercises
- ☐ General exercise (e.g. walking)
- ☐ Advice to stay active
- ☐ Advice regarding alternate ways of moving or performing activities
- ☐ Advice to avoid pain provoking movements
- ☐ Work modification
- ☐ Spinal manipulation/adjustment
- ☐ Mobilisation
- ☐ Massage
- ☐ Lumbar supports
- ☐ Spinal traction
- ☐ Acupuncture
- ☐ Electrotherapy (e.g. TENS, interferential, ultrasound)
- ☐ Thermal modalities (e.g. heat, ice)
- ☐ Printed information (*please specify*) ___________________________
- ☐ Other 1 (*please specify*) ___________________________
- ☐ Other 2 (*please specify*) ___________________________
- ☐ Other 3 (*please specify*) ___________________________
- ☐ Referral to another health care provider (e.g. another chiropractor, general practitioner, pain clinic, specialist).
  If yes, please specify ___________________________

If you wish to comment on your management decisions, please do so here:

